# Reporting quality of sham needles used as controls in acupuncture trials: a methodological evaluation

**DOI:** 10.1186/s13020-022-00608-5

**Published:** 2022-05-31

**Authors:** Ye-Seul Lee, Song-Yi Kim, Mariah Kim, Minseo Kim, Jiyoon Won, Hyangsook Lee, Myeong Soo Lee, Younbyoung Chae

**Affiliations:** 1 Jaseng Spine and Joint Research Institute, Jaseng Medical Foundation, Seoul, Republic of Korea; 2 Department of Anatomy and Acupoint, College of Korean Medicine, Gachon University, Seongnam, Republic of Korea; 3 Department of Internal Medicine, Korean Medicine Hospital of Pusan National University, Yangsan, South Korea; 4 KM Science Research Division, Korea Institute of Oriental Medicine, Daejeon, South Korea; 5 Department of Science in Korean Medicine, College of Korean Medicine, Graduate School, Kyung Hee University, Seoul, South Korea; 6 Acupuncture and Meridian Science Research Center, College of Korean Medicine, Kyung Hee University, 1 Hoegi-dong, Dongdaemun-gu, Seoul, Republic of Korea

**Keywords:** Placebos, Acupuncture, Control, STRICTA, CONSORT, TIDieR

## Abstract

**Objective:**

The description of controls is important in acupuncture clinical trials to interpret its effectiveness without fallacy. This paper aims to evaluate the reporting quality of acupuncture studies on the characteristics of sham needles.

**Study design and setting:**

Using a checklist developed from previously published reporting guidelines, the distribution of reported items and changes of reporting rates over time were investigated. Two-way ANOVA and linear regression were conducted.

**Results:**

Original articles of RCTs of any design involving sham needles as controls were eligible for assessment. 117 trials from three 2-year time periods between 2009 and 2018 were included. Seven items out of 25 were reported in more than 50% of the studies. While significant differences of reporting scores among categories were observed, there were no significant differences among time periods; no significant improvement was observed over time.

**Conclusions:**

Low reporting qualities of sham needles used in acupuncture studies may influence how researchers understand the effectiveness of acupuncture. This study evaluated previous publications from 2009 to 2018 and found that reporting qualities on sham needles did not improve over time. Further studies are required to validate the items used in this study to endorse better reporting of controls in acupuncture trials.

## Introduction

Acupuncture is an intervention used for therapeutic purposes performed by “inserting one or more needles into specific sites on the body surface for therapeutic purposes” [1]. The characteristics of acupuncture needles need to be considered in the overall design of clinical trials, in specific, in the selection of controls, to interpret the effect of acupuncture needles without fallacy. Placebo controls must be hard to distinguish from real acupuncture needles without producing any physiological therapeutic effect [2]. In this regard, non-penetrating placebo needles have been used as placebo controls to verify the efficacy of acupuncture in clinical trials [3, 4]. Sham needles exist in several forms including the Streitberger, the Park, and the Takakura needles, and have been employed in acupuncture trials for decades.

Despite the development and application of sham needles, however, the assumption that components of sham needles are inactive or inert can be misleading. A number of trials reported conflicting results regarding the effectiveness of acupuncture [5]. Several studies have shown that the effectiveness of sham needles is similar to that of real acupuncture needles [6–8], and the validity of sham needles as controls in acupuncture research has been argued for several years [9, 10]. Some of the recent works imply unintended physiological effects of placebo needles that may not always be accounted for when calculating the specific effects of acupuncture needles [2, 10, 11]. While reviews have been published to address this contradictory results in acupuncture trials [5, 9], the focus of these reviews was mostly on the choice of placebo devices in terms of credibility in blinding and effectiveness as controls.

A recent study implied that characteristics of the placebo device may influence the apparent effectiveness of the active intervention, and that adequate description of placebo device may be the solution to this problem [12]. Guidelines related to acupuncture trials that are previously published and in use include “CONsolidated Standards Of Reporting Trials” (CONSORT) statements [13, 14], the “STandards for Reporting Interventions in Controlled Trials of Acupuncture” (STRICTA) guidelines as extensions of CONSORT [15, 16], and the “Template for Intervention Description and Replication” (TIDieR) checklist as another extension of CONSORT item 5 (“Intervention” in the “Methods” section) [17]. Previous studies investigating the reporting qualities of the clinical trials before and after the publication of guidelines showed that the development of CONSORT [18, 19] and STRICTA [20, 21] led to improvements of the overall reporting qualities. Cross-sectional investigations on the quality of intervention reporting of clinical trials based on TIDieR checklist [22–25] showed that while average completion of the checklist items did not show significant changes, the evaluations allowed further understanding of the description of interventions in clinical trials. However, the description of sham needles used in acupuncture trials have been partially addressed, suggesting that controls be described in a similar manner to active intervention. STRICTA, for instance, suggests in a single item that the rationale and description of the controls be described, and details to be provided if sham needle is used in the trial [15, 16].

To date, there has been no formal systematic evaluation of whether sham needles are reported without missing information in clinical trials. Without adequate description of sham needles, the clinical effectiveness of acupuncture needles reported in clinical trials are open to miscalculation and misinterpretation. A process of assessment by the authors of the CONSORT and STRICTA have been proposed and published [18, 20, 21]. By adopting a similar process of evaluation for sham needles used in randomized clinical trials (RCTs), this study aimed to systematically analyze the reporting quality of the sham needles in acupuncture trials.

## Methods

### Study design

A methodological evaluation of the reporting qualities of placebo needles in acupuncture trials was conducted in this study. The methodological basis was a systematic review adjusted to allow focus on reporting quality of the controls in published articles. Methodologies in previous studies investigating the reporting quality of acupuncture trials before and after the publication of STRICTA were partially adopted to provide a basis of comparison between the overall reporting quality of acupuncture trials and quality of reporting sham needles from the same time periods [20, 21].

### Time periods and eligibility criteria

To track changes over time, three distinct two-year time periods were selected. As a baseline, time period 2009–2010 was selected to evaluate the reporting quality around the publication date of the revised version of STRICTA and CONSORT [13, 15]. Two of the time periods, or period 2009–2010 and 2014–2015 were matched with previous studies [20, 21] to allow direct comparison of the overall reporting qualities of published acupuncture trials. The last time period 2017–2018 was selected to reflect the recent reporting qualities of published articles.

The eligibility criteria included original articles of RCTs of any design involving sham needles as controls on humans, in English language between the above date ranges. Since this review aimed to evaluate the reporting quality of trials using sham needles, we excluded trials using interventions such as electroacupuncture, laser acupuncture, pharmacopuncture, transcutaneous electrical nerve stimulation (TENS), acupressure, and trials which use acupuncture needles but do not use sham needles as controls. Abstracts in conference proceedings and protocols without results from the trials were also excluded. PubMed (https://pubmed.ncbi.nlm.nih.gov/) was searched for this study.

### Development of the evaluation checklist

A reporting quality assessment checklist was developed for this study involving 25 items for trials incorporating acupuncture control group using sham needles (Table 1). Items were based on STRICTA, CONSORT and TIDieR, which were closely worded to the original recommendations. Six categories of the checklist include: (I) Type of placebo needle, (II) Details of sham needling, (III) Location of sham needling, (IV) Treatment regimen, (V) Practitioner, and (VI) Protocol and settings. In addition, new items were added based on the updates on the placebo effects of sham needles investigated in previous studies. The newly added items focused on the interactions between the practitioner and patient, and therefore are found in categories (V) Practitioner and (VI) Protocol and settings. Examples of these items include whether practitioner and patient had discussions prior to or during the treatment [2, 26–29], introduction of the treatments and instructions given to patients [30–34], and method of blinding [35–38]. All of the items were phrased as a series of questions to which the answer could be given logistically as a “yes” or “no,” and each item was scored based on the evaluated answers.

**Table 1:** Items used in this study for systematic evaluation

Items			Corresponding items in guidelines	
Categories		Descriptions	STRICTA	TIDieR
I. Type of sham needle	1a	Type of sham needle. (e.g., Streitberger needle, Park needle, Takakura needle, cocktail picks)	1a	1
	1b	Rationale for using the chosen sham needle	1b, 6a	2
II. Details of sham needle	2a	Number of sham needling per subject per session	2a	8
	2b	Are the depths of insertion reported?	6 g	
	2c	Response sought (e.g., de qi or muscle twitch response)	2d	6
	2d	Sham needle stimulation	2e	6
	2e	Sham needle retention	2f	6
	2f	Details of other interventions administered in addition to sham needles	4a	6
III. Location of sham needle	3a	Location of sham needling (e.g., acupoints/non-acupoints, exact location of the sites)	2b	6
	3b	Is it explicit that the points are unilateral or bilateral?	6e	6
	3c	Rationale for the location chosen for sham needling	1b, 6a	2
IV. Treatment regimen	4a	Number of placebo sessions	3a	8
	4b	Frequency and duration of placebo sessions	3b	8
	4c	Total trial period for placebo group/sessions	3a, 3b	
V. Practitioner	5a	Is the same practitioner administering both treatment and control groups?		5
	5b	Did practitioner and the patient have discussions prior to the treatment? (Doctor-patient relationship)		
	5c	Was there any discussion regarding the symptoms during the treatment?		
VI. Protocol and settings	6a	Instruction and information given by the practitioner to the patients (are the explanations that were given to participants of treatment and control interventions provided?)	6b	3
	6b	Was the introduction to participants explained in the paper?		
	6c	Did the instruction and information include the term “placebo” or “sham"?		
	6d	Type of blinding (e.g., only the patients, double-blinding)		
	6e	Was the method of blinding sham needle to patients elaborated?		
	6f	Modification of the needling procedure if there was any, and reason for the modification		10
	6 g	Assessment of the intervention adherence or fidelity, and blinding		12
	6 h	Any differences in the settings between treatment and control groups		

### Data selection and screening process

After manual removal of duplicates from the records initially searched through PubMed, articles were screened based on the eligibility criteria described above. Prior to the assessment of the trials, three assessors (MK1, MK2, YSL) discussed the meaning of each item in detail, and went through a pilot assessment using an article that was not included in the final review. When the meaning of an item was understood differently among the assessors, the wording of the criteria was revised to reach a consensus. For the final review, two reviewers (MK1 and MK2), both experienced researchers in acupuncture, assessed each article independently. A third reviewer (YSL) compared the two evaluations and checked the differently evaluated items. Reviewers MK1 and MK2 returned to the evaluations and discussed the differently marked items until they reached an agreement. Meanwhile, the third reviewer (YSL) assessed the studies independently. Once the evaluations agreed between the first two reviewers (MK1 and MK2) were completed, the third reviewer (YSL) compared the evaluations again and checked the differently evaluated items. The final agreed evaluations among the three reviewers were selected as the final mark of each item.

### Primary outcomes

Outcome measures for this study were the item scores using the checklist described above. After the evaluation was completed, the scores of each item were summarized using logistic criteria. All items were given equal weight such that each item contributed a score of 0 or 1.

### Statistical analysis

The mean of the percentage of reported items were analyzed by categories and in total. Data were summarized for each time period. Two-way analysis of variance (ANOVA) comparing time periods and categories was conducted to observe significantly different scores. To investigate which group means are different, post hoc comparisons were analyzed between different time periods by categories using Tukey’s honestly significant difference (HSD) test. Furthermore, linear regression analysis was conducted between the published date of the individual studies and overall item scores to observe the trend of the reporting quality of sham needles over time. All statistical analyses were conducted using R (version 4.0.4, The R Foundation for Statistical Computing).

## Results

### Study characteristics

**Fig. 1:**
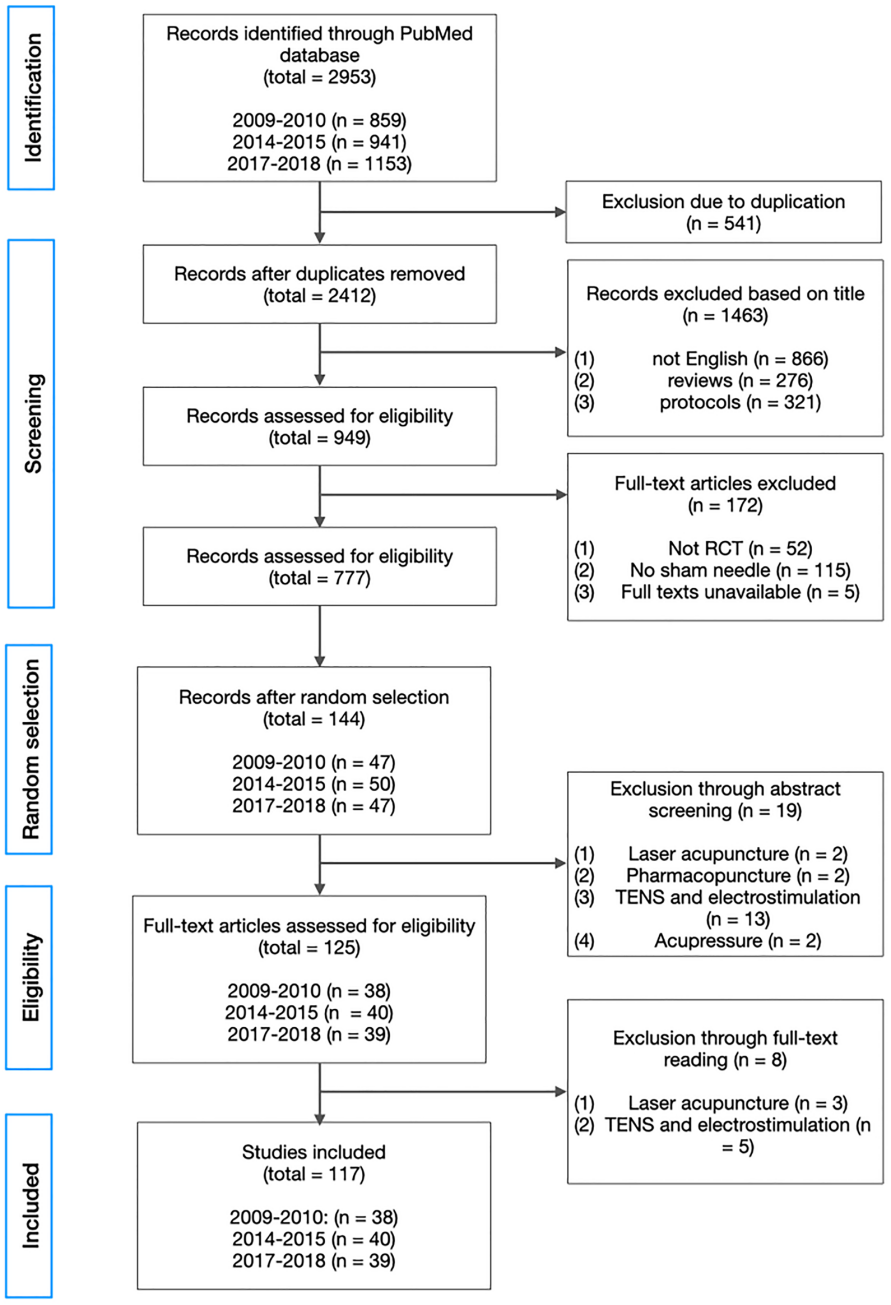
PRISMA (preferred reporting items for systematic reviews and meta-analyses) flowchart showing the flow of articles through the study

From the initial search through PubMed, a total of 2953 studies were identified (Fig. 1). After manual removal of duplicates, 2412 articles were screened based on title. Non-English articles, review articles, and protocol articles were removed from the record. After title screening and full-text obtaining, total of 144 articles were randomly selected from three time periods for full-text reading. 27 articles were removed upon abstract screening and full-text reading for using interventions outside the scope of this review (i.e., laser acupuncture, pharmacopuncture, TENS or electrostimulation, and acupressure). As a result, a total of 117 articles were included (Fig. 1).

### Types of reported sham controls

Among the 117 papers evaluated in this study, a total of 50 studies (43%) mentioned the type of sham needles. Among these 50 papers which reported the specific type of sham needles, the most commonly reported sham needle was Streitberger placebo needle (14 studies), followed by Park sham needle (8 studies). A number of papers described sham needles in a general expression such as “blunt tip needle” or “non-penetrating needle” (8 studies), and a few studies used needle handles (2 studies). Shallow needling or minimal insertion was reported in 6 studies, and real needle on non-acupoints was reported in 3 studies. Takakura needle and sham laser device were reported in 2 studies, respectively. Other types of reported sham devices were specific devices developed by the authors and Kim sham needle.

### Differences in reporting quality among categories

**Fig. 2:**
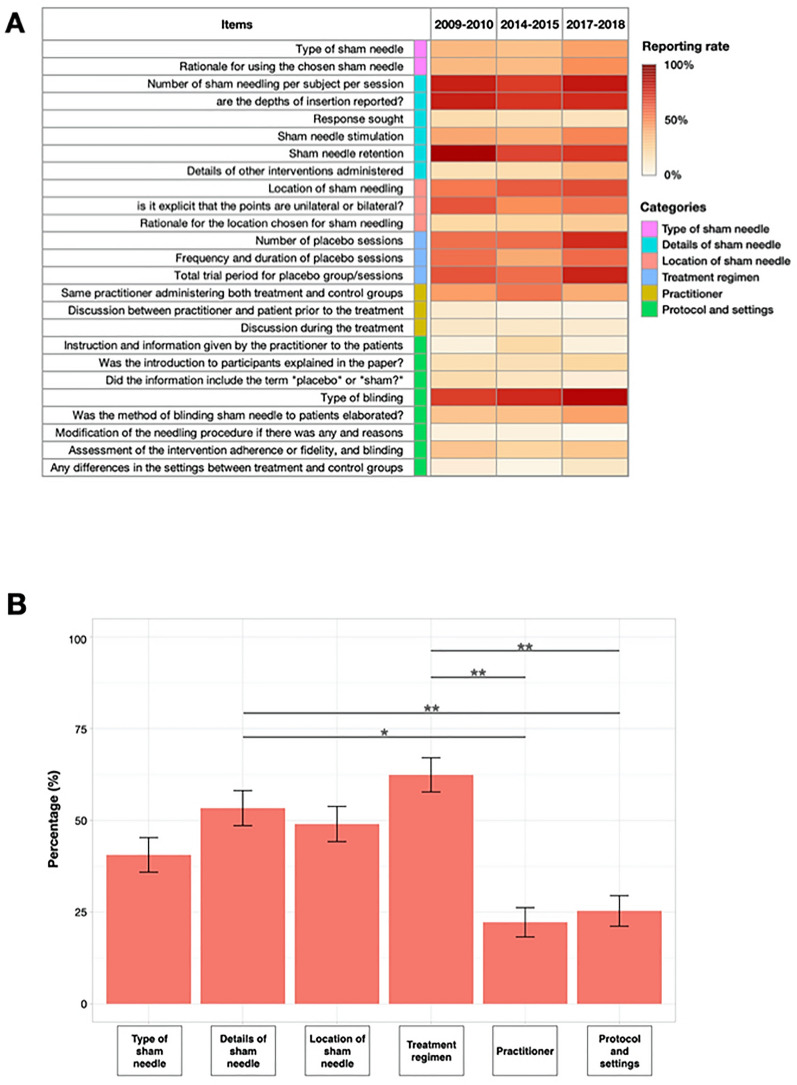
**A **Heatmap showing the distribution of reporting rates in each time period by each item. Items in “treatment regimen” category were reported in more than 50% of the studies throughout all time periods, and items in “practitioner” category and “protocol and settings” category were reported in less than 50% of the studies. **B** Bar graph showing the overall reporting scores by categories. The most reported category was “treatment regimen”, and the least reported category was “practitioner.” *p-value < 0.05. **p-value < 0.01

Overall, a total of 7 items out of 25 were reported in more than 50% of the studies throughout all time periods: number of placebo needling, depths of insertion (including non-penetration), placebo needle retention, location of placebo needling, number of treatment sessions, total trial period, and blinding. Observing by categories, only the items in “treatment regimen” category were reported in more than 50% of the studies throughout all time periods. On the other hand, items in “practitioner” category and “protocol and settings” category were seldom reported and the frequency percentage was estimated to be far below 50% (Fig. 2A). Two-way ANOVA showed significant difference of reporting scores among categories [F(5, 57) = 5.992, *p*-value < 0.001]; however, no significant difference in reporting scores among time periods [F(2, 57) = 0.291, *p*-value = 0.749], and there was no significant interaction between the effects of time period and categories [F(10, 57) = 0.109, *p*-value = 1.000]. Post-hoc comparisons using Tukey HSD test indicated that the mean reporting score of the category “details of sham needle” was significantly higher than “practitioner” (mean difference = − 0.31, adjusted p-value = 0.022) and “protocol and settings” (mean difference = − 0.28, adjusted p-value = 0.004). Likewise, the mean reporting score of the category “treatment regimen” was significantly higher than “practitioner” (mean difference = − 0.40, adjusted p-value = 0.007) and “protocol and settings” (mean difference = − 0.37, adjusted p-value = 0.002) (Fig. 2B).

### Changes of reporting quality over time

**Fig. 3:**
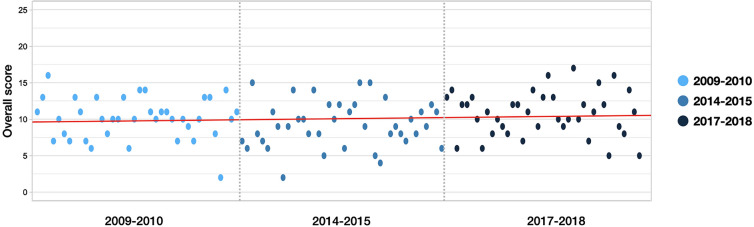
Distribution of the overall reporting scores over time. The red line shows the linear regression value between published date and reporting scores (β = 0.008, p-value = 0.358). The reporting qualities on sham needles did not improve over time

Regression analysis between the overall scores of the papers and time showed that there was no statistically significant change of overall item scores (β = 0.008, p-value = 0.358). Average score of the studies published in period 2009–2010 was 10.03, 9.45 in period 2014–2015, and 10.71 in period 2017–2018 (Fig. 3). Observing by items, a constantly increasing trend over time were observed in items including “number of sham needling”, “location of sham needling”, “number of treatment sessions”, “frequency and duration of treatment”, “total trial period”, and “blinding”.

## Discussion

This study evaluated the reporting quality of sham needles in acupuncture trials, in three time periods between the year 2009 and 2018, using systematic methodology adopted and modified from previous studies [18, 20, 21]. For this systematic evaluation, a checklist was developed based on CONSORT, STRICTA, TIDieR, and previous literature investigating placebo effects in acupuncture treatments. While significant differences of reporting scores among categories were observed, there were no significant differences among time periods; similarly, no significant improvement of reporting scores was observed over time. To our knowledge, this is the first paper to systematically analyze the reporting quality of sham needles used in acupuncture trials.

The results showed that overall, a total of 7 items out of 25 were reported in more than 50% of the studies throughout all time periods. These items include: “number of sham needling”, “depths of insertion (including non-penetration)”, “sham needle retention”, “location of sham needling”, “number of treatment sessions”, “total trial period”, and “blinding”. Only the “treatment regimen” category was reported in more than 50% of the studies throughout all time periods. Items which showed a constantly increasing trend over time and were above 50% reporting rate were: “number of sham needling”, “location of sham needling”, “number of treatment sessions”, “frequency and duration of treatment”, “total trial period”, and “blinding”, implying a continuously increasing trend of reporting the above information in published literature. On the other hand, items which showed a constantly decreasing trend over time and were below 50% reporting rate were: “response sought for sham needle (e.g., de qi, muscle twitch)”, “whether the practitioner and the patient have any interaction during the trial”, “whether there was any discussion regarding the symptoms”, and “information include the term ‘placebo’ or ‘sham’.”

Modified items from STRICTA such as “type of sham needle” were not reported in more than 50% of the studies throughout all time periods, while a previous study found significantly increased reporting of the “specific style of acupuncture,” the corresponding item in STRICTA, over time since the publication of the checklist [21]. Moreover, modified items from TIDieR such as “modification of the needling procedure” was one of the least reported items. Previous studies on the impact of STRICTA and CONSORT on reporting qualities of acupuncture trials showed an increased quality of reporting since the publication of STRICTA in 2010 [18, 20, 21]; in contrast, the results from this study illustrated that reporting quality of sham needles did not improve over time.

Furthermore, items that were not listed in either STRICTA, CONSORT or TIDieR but nonetheless discussed in recent studies to have substantial impact on the placebo effect by sham needles, were reported in very few studies. These items include discussions between practitioner and patients before and during the treatments, information given to patients, and confirmation of whether the information given to patients included the term “sham” or “placebo.” Previous studies discussed the importance of the doctor-patient relationship in placebo effects [26–29]; recent studies also point out that acupuncture treatment, with its long duration of time for the application of the needles and the communication involved, consequently builds a stronger doctor-patient relationship which may lead to stronger placebo effects [2, 39]. While it would be nonsensical to report all types of communications during the trial, the scope of communications between the practitioner and the patient, i.e., whether the conversation was carried out beyond the scope of the experiment, and whether any kind of interaction to build a trusting relationship between the practitioner and the patient was allowed, would enable the readers to understand the potential non-specific effects involved in the process of the treatment.

On the other hand, literature discuss that the extent of disclosure of information may influence the level of placebo effect on patients [30, 32–34]. One study showed that the placebo responses caused by information disclosure modifies the drug response [31]. Lastly, blinding is an important aspect of controlled trials [36, 38]. It is also found to be extremely difficult to achieve in acupuncture trials [35, 37], which requires caution in both the experiment design and the reporting of the experiment.

The findings in this study showing low rates of reporting information about sham needle and its treatment protocol would most likely lead to a gap of reporting qualities between that of acupuncture in general and that of sham needles. Previous studies presented that reporting qualities of acupuncture RCTs improved overall since the publication of STRICTA and CONSORT [18, 20]. One study showed that the best-reported item was adhered to in over 90% while poorest reporting value was 51.1% [18, 20, 21]. In contrast, this study showed that only seven items out of 25 were reported in more than 50% of the studies. Similarly, the overall trend of reporting qualities based on STRICTA was shown to constantly improve in the previous studies [20, 21], while the reporting qualities of sham needles reviewed in this study did not show significant improvement over time.

Low reporting qualities of the controls used in acupuncture trials compared to reporting qualities of the intervention in general puts at risk the accurate analysis of specific effect of acupuncture; and this may require a reappraisal of the current understanding of the effectiveness of acupuncture. As with other types of medical interventions investigated in RCTs, the specific effects of acupuncture have often been analyzed through contrast of the reported effectiveness of the treatment to that of control. Therefore, the data presented in this study might potentially imply that the specific effects of acupuncture might have been misinterpreted due to missing information regarding controls. Furthermore, research such as meta-analyses and systematic reviews based on literature, might have even higher chance of misunderstanding of the effectiveness of acupuncture, simply due to the accumulation of fallacies over time. It may not be pure coincidence that placebos in acupuncture trials have been subject to strong effectiveness, sometimes as effective as verum acupuncture, by a number of researchers for decades [6, 39–43], and might be due to the lack of elaboration on controls used in acupuncture trials.

The limitations of this study include that the scoring system based on the checklist to evaluate reporting qualities. STRICTA, CONSORT and TIDieR were never intended to be employed as rating scales [21, 44, 45], and the items in the checklist used in this study were mostly adopted from these guidelines. It is important to emphasize that the score of each paper does not reflect the quality of the research itself. Furthermore, the items in this study were equally weighted based on previous researches [20, 21, 45], which may be subject to further discussion. However, despite these limitations, this study allowed a systematic evaluation of the reporting qualities of the previous researches using sham needles, and allowed a comparison of the results with the reporting qualities of acupuncture trials investigated in previous studies by adopting the time span as well as the items. The systematic methodology used in this study allowed for an objective evaluation of the studies by multiple reviews and discussions.

In conclusion, we found that the reporting qualities on sham needles did not improve over time. Many of the items were reported in less than 50% of the evaluated studies. In contrast to the reporting quality of acupuncture trials in general as reported in previous studies, low reporting qualities regarding controls may influence how researchers understand the effectiveness of acupuncture. Further studies are required to validate the items used in this study to endorse better reporting of sham needles used as controls in acupuncture trials.

## Conclusions

This study evaluated the reporting quality of sham needles in acupuncture trials. Evaluation of previous publications from 2009 to 2018 showed that reporting qualities on sham needles did not improve over time. Significant differences of reporting scores among categories were observed, while no significant differences were found among time periods. Low reporting qualities of sham needles used in acupuncture studies may influence the understanding of the effectiveness of acupuncture. Further studies are required to validate the items used in this study to endorse better reporting of controls in acupuncture trials.

## Data Availability

The data and the related materials are available upon request to the first author.

## Abbreviations

CONSORT: CONsolidated Standards Of Reporting Trials
STRICTA: STandards for Reporting Interventions in Controlled Trials of Acupuncture
TIDieR: Template for Intervention Description and Replication
RCTs: Randomized Clinical Trials
TENS: Transcutaneous Electrical Nerve Stimulation
ANOVA: Analysis of variance
HSD: Honestly Significant Difference
PRISMA: Preferred Reporting Items for Systematic reviews and Meta-Analyses
